# Electrochemical Reduction of CO_2_ to Oxalic Acid: Experiments, Process Modeling, and Economics

**DOI:** 10.1021/acs.iecr.2c02647

**Published:** 2022-09-28

**Authors:** Vera Boor, Jeannine E. B. M. Frijns, Elena Perez-Gallent, Erwin Giling, Antero T. Laitinen, Earl L. V. Goetheer, Leo J. P. van den Broeke, Ruud Kortlever, Wiebren de Jong, Othonas A. Moultos, Thijs J. H. Vlugt, Mahinder Ramdin

**Affiliations:** † Engineering Thermodynamics, Process & Energy Department, Faculty of Mechanical, Maritime and Materials Engineering, Delft University of Technology, Leeghwaterstraat 39, 2628 CB Delft, The Netherlands; ‡ Department of Sustainable Process and Energy Systems, TNO, Leeghwaterstraat 44, 2628 CA Delft, The Netherlands; ¶ VTT Technical Research Centre of Finland, Tietotie 4 E, Espoo 02044, Finland; § Large-Scale Energy Storage, Process & Energy Department, Faculty of Mechanical, Maritime and Materials Engineering, Delft University of Technology, Leeghwaterstraat 39, 2628 CB Delft, The Netherlands

## Abstract

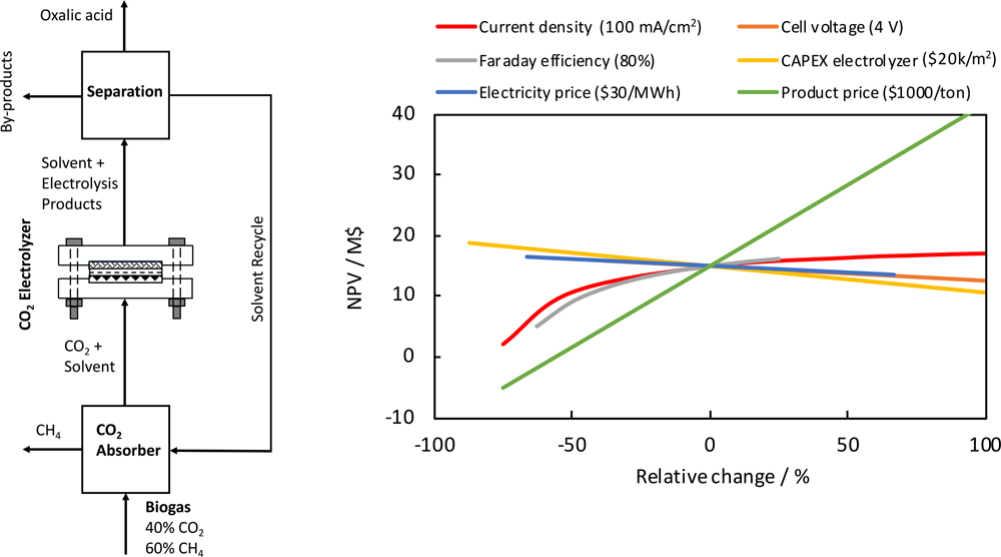

We performed H-cell and flow cell experiments to study the electrochemical reduction of CO_2_ to oxalic acid (OA) on a lead (Pb) cathode in various nonaqueous solvents. The effects of anolyte, catholyte, supporting electrolyte, temperature, water content, and cathode potential on the Faraday efficiency (FE), current density (CD), and product concentration were investigated. We show that a high FE for OA can be achieved (up to 90%) at a cathode potential of −2.5 V vs Ag/AgCl but at relatively low CDs (10–20 mA/cm^2^). The FE of OA decreases significantly with increasing water content of the catholyte, which causes byproduct formation (e.g., formate, glycolic acid, and glyoxylic acid). A process design and techno-economic evaluation of the electrochemical conversion of CO_2_ to OA is presented. The results show that the electrochemical route for OA production can compete with the fossil-fuel based route for the base case scenario (CD of 100 mA/cm^2^, OA FE of 80%, cell voltage of 4 V, electrolyzer CAPEX of $20000/m^2^, electricity price of $30/MWh, and OA price of $1000/ton). A sensitivity analysis shows that the market price of OA has a huge influence on the economics. A market price of at least $700/ton is required to have a positive net present value and a payback time of less than 10 years. The performance and economics of the process can be further improved by increasing the CD and FE of OA by using gas diffusion electrodes and eliminating water from the cathode, lowering the cell voltage by increasing the conductivity of the electrolyte solutions, and developing better OA separation methods.

## Introduction

Oxalic acid (OA) is an important base chemical that is mainly used for metal treatment, textile treatment, concentration of rare earth elements, bleaching, and chemical synthesis. OA has been proposed as a feedstock to produce sustainable polyester, which is a polymer with a multibillion dollar market size.^1^ Currently, OA is predominantly produced from the oxidation of carbohydrates, olefins, and CO. All three methods require multiple complicated processing steps involving high pressure and/or temperature conditions and acid/base consumption.^2^ A more recent approach of producing oxalic acid is based on the electrochemical reduction of CO_2_ according to the half-cell reaction^3,4^
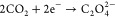
1We note that oxalate formation may involve different reaction steps, including initial electron transfer and radical–radical dimerization of CO_2_. On lead (Pb) or mercury (Hg) electrodes, oxalic acid is the major product in nonaqueous solvents, but in the presence of water it can further be reduced to higher carboxylic acids like glyoxylic acid (GOA) and glycolic acid (GCA):^5^
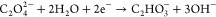
2
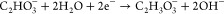
3Carbon monoxide (CO) can also be produced in nonaqueous solvents according to the half-cell reaction:^6^
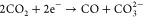
4Note that CO formation may proceed through several intermediate steps, which are not shown here.^3,6^ CO_2_ reduction on the OA-producing electrodes (i.e., Pb or Hg) in aqueous solvents or nonaqueous solvents with a sufficiently high water concentration shifts the mechanism from oxalate to formate:^7,8^
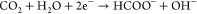
5The past decade, electrochemical reduction of CO_2_ has been studied intensively but mostly in aqueous solvents.^9^ Data on CO_2_ reduction in nonaqueous solvents is relatively scarce despite the well-known advantages of these solvents such as high CO_2_ solubility and suppression of the competing hydrogen evolution reaction (HER).^10^ A compilation of literature studies on oxalic acid/oxalate production from electrochemical CO_2_ reduction can be found in Table S1 of the Supporting Information. From this overview, it is clear that oxalate can be obtained in nonaqueous solvents with a high Faraday efficiency (FE) but at relatively low current densities (CD  100 mA/cm^2^).^11−19^ Most of these experiments were performed in the liquid phase in an H-cell type of reactor, which results in low current densities due to mass transfer limitations. Recently, König et al.^19^ used a Pb gas-diffusion electrode (GDE) in a flow cell (flow-through configuration) to convert CO_2_ to oxalate with an FE of 53% at a CD of 80 mA/cm^2^. These authors observed catalyst breakdown at high CDs (100 mA/cm^2^) due to cathodic corrosion of Pb in the presence of tetraalkylammonium salts at high negative potentials. Electrochemical reduction of CO_2_ to oxalate in nonaqueous solutions appears to be more challenging than other electroreduction products like formic acid (FA), CO, and hydrocarbons, which have been produced with high FEs and CDs (1 A/cm^2^) in aqueous solutions.^20−23^ As we will see later, the challenges for CO_2_ reduction to oxalate are related to finding proper catalysts, electrolytes, and membranes for stable operation in nonaqueous solvents and downstream separation of products. It is noteworthy to mention that Marx et al.^24^ recently revisited CO_2_ reduction to oxalate with first-row transition metal complexes and concluded that several previously published works are irreproducible, lack sufficient analysis, and report misleading analytical data and conflicting reactivity.

In this work, we studied the electrochemical reduction of CO_2_ to oxalic acid in nonaqueous solvents using a Pb catalyst. An H-cell was used to investigate the effects of anolyte, catholyte, supporting electrolyte, temperature, water content, and cathode potential on the performance indicators (i.e., FE, CD, and product concentration). The best conditions of these screening experiments were selected to study the CO_2_ electrolysis performance in a flow cell setup. In addition, we assessed the technical and economic feasibility of oxalic acid production from the electrochemical conversion of CO_2_. A process design including CO_2_ capture, electrochemical conversion, and downstream processing of oxalate is presented. The effects of different parameters (i.e., FE, CD, cell voltage, electricity price, product concentration, and electrolyzer capital cost) on the net present value (NPV) and payback time (PBT) are investigated.

The manuscript is organized as follows. In the next section, we will discuss the experimental details for CO_2_ electrolysis in the H-cell and flow cell setups. In a subsequent section, the experimental results for both the H-cell and the flow cell setups will be presented. We will present the process design and modeling details for the electrochemical conversion of CO_2_ to oxalic acid including CO_2_ capture, CO_2_ electrolysis, and downstream separation. In the penultimate section, the details and results of the economic analysis will be presented. In the final section, the main conclusions of this work will be summarized.

## Experimental Section

The CO_2_ electrolysis experiments were performed in two setups (H-cell and flow cell). The cell configuration and settings of both setups will be discussed next.

### H-Cell Measurements

For all experiments, reagent grade chemicals were purchased from Sigma-Aldrich and used as received. The glassware was thoroughly cleaned by storing it overnight in a KMnO_4_ solution, washing it with a 0.1 M H_2_O_2_ solution followed by a wash with deionized water, and rinsing the cell components with acetone to remove residual water. The cell was composed of a platinum wire with a surface area of 10 cm^2^ as the anode, a cation exchange membrane (CEM, Nafion-117 from Fumatech), a Pb wire (Alfa Aesar, 99.9%) with a surface area of 10 cm^2^ as the cathode, and a leak-free Ag/AgCl reference electrode (Inovative Instruments LF-1-100) situated in the cathode compartment. As catholyte, propylene carbonate (PC) with 0.7 M tetraethylammonium chloride (TEACl) was used. Three different types of supporting electrolytes were tested (i.e., TEACl, tetraethylammonium acetate (TEAA), and tetrabutylammonium perchlorate (TBAP)). As anolyte, an aqueous solution with 0.5 M H_2_SO_4_ or ACN with 0.1 M TEACl was used. The catholyte was saturated with CO_2_ by bubbling with a flow rate of 18 L/h for 1 h. The amount of anolyte and catholyte in each compartment was ca. 160 mL. The Pb electrode was pretreated by shortly applying −1.8 V vs Ag/AgCl in a 0.5 M H_2_SO_4_ solution. The experiments were performed in potentiostatic mode for 5 h. The liquid products in the cathode compartment were analyzed every 30 min with high performance liquid chromatography (HPLC, Agilent). The gaseous products from the cathode were not analyzed. The water content in the catholyte was measured with a Karl Fischer (KF) titrator. The applied cathode potential, the temperature, and the types of anolyte, catholyte, and supporting electrolyte were varied in the experiments.

### Flow Cell Measurements

For the flow cell measurements, a similar cleaning, washing, and pretreatment procedure was applied as in the H-cell experiments. A Pb plate (Alfa Aesar, 99.9%) and a Pt wire, both with a surface area of 10 cm^2^, were used as a cathode and anode, respectively. The cathode and anode compartments were separated with a Nafion-117 membrane. To control the working potential, a leak-free Ag/AgCl reference electrode was used in the cathode compartment. PC with 0.7 M TEACl or 0.3 M TBAP and 0.5 M H_2_SO_4_ were used as catholyte and anolyte, respectively. Both the catholyte and anolyte were pumped through the cell with a flow rate of 3.6 L/h/cm^2^. The CO_2_ electrolysis experiments were performed in the potentiostatic mode for 4.5 h. The liquid products were sampled every 45 min and analyzed with HPLC. The gaseous products were not analyzed. The water content in the catholyte was measured with KF titration.

## Experimental Results

**Figure 1:**
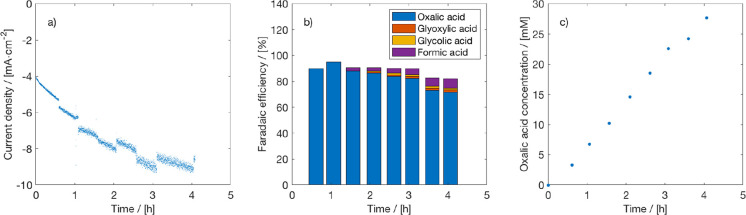
Time dependent electrolysis of CO_2_ to oxalate. (a) Current density, (b) Faraday efficiency, and (c) OA concentration for electrochemical reduction of CO_2_ on a Pb cathode in PC with a 0.7 M TEACl supporting electrolyte at −2.5 V vs Ag/AgCl in an H-cell at 298.15 K. A Pt anode, 0.5 M H_2_SO_4_ as anolyte, and CEM (Nafion 117) were used.

In Figure 1, the results of H-cell experiments of 4 h of CO_2_ electrolysis on a Pb cathode in PC with 0.7 M TEACl as supporting electrolyte at −2.5 V vs Ag/AgCl are shown. Clearly, the CD, byproduct formation, and OA concentration increased as a function of time. The FE of OA decreased from around 90% to 70%, while the CD increased from 4 mA/cm^2^ to ∼10 mA/cm^2^. The FE of the liquid byproducts (formic acid, glycolic acid, and glyoxylic acid) increased over time. This is likely due to an increase in the water content of the catholyte, since the byproducts are only formed in the presence of water. The transportation of water from the anode to the cathode may occur due to diffusion and electro-osmotic drag (EOD). Some (uncharacterized) gaseous byproducts are formed as well, because the FE of the liquid products is lower than 100%. The concentration of OA increased to ∼30 mM due to the recycling of the catholyte. Experimental results at different cathode potentials (−2.2, −2.3, −2.4, and −2.7 V vs Ag/AgCl) showed similar trends and can be found in Figures S1–S4 of the Supporting Information.

**Figure 2:**
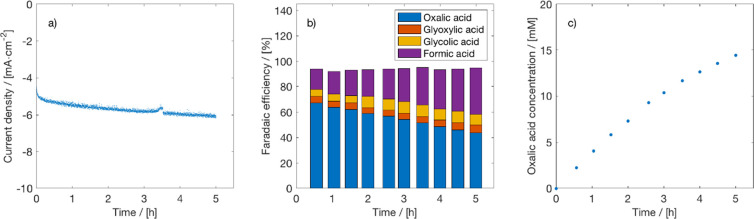
Effect of water on CO_2_ electrolysis to oxalate. (a) Current density, (b) Faraday efficiency, and (c) OA concentration for electrochemical reduction of CO_2_ on a Pb cathode in PC with 0.7 M TEACl supporting electrolyte and 1 vol % water at −2.5 V vs Ag/AgCl in an H-cell at 298.15 K. A Pt anode, 0.5 M H_2_SO_4_ as anolyte, and CEM (Nafion 117) were used.

The effect of water on the FE was tested by performing experiments under conditions similar to those used previously but now in a catholyte that contained 1 vol % water. The results can be seen in Figure 2, which confirms that the presence of water significantly reduces the FE of OA, while promoting the formation of byproducts. This means that the catholyte should be kept water-free during the electrolysis process, but this is not an easy task as long as water is oxidized at the anode. We note that it is possible to have an alternative oxidation reaction at the anode (e.g., hydrogen oxidation) to limit the crossover of water.

The effect of different catholytes and anolytes on OA production was tested in the H-cell setup. The results of CO_2_ electrolysis on a Pb cathode in acetonitrile (ACN) with 0.1 M TEACl supporting electrolyte at −2.5 V vs Ag/AgCl are shown in Figure S5 of the Supporting Information. In these experiments, the anolyte was 0.5 M H_2_SO_4_. Compared to PC, the use of ACN as the catholyte resulted in more byproduct (mainly formic acid) formation and lower OA concentrations (∼4 mM after 5 h) at similar CDs. The FE of OA at the start of the experiment was 50% but dropped to 10% after 5 h of experiments. The lower FE of OA in AN, relative to PC, is likely due to a higher diffusion rate of water in the former. These results are in agreement with the observations of Hori.^5^ Subsequently, we changed the anolyte from 0.5 M H_2_SO_4_ to ACN with 0.1 M TEACl, while keeping the same catholyte (0.1 M TEACl in ACN). Note that in this case ACN is oxidized at the anode, which is not desired as ACN is expensive. The results in Figure S6 of the Supporting Information show that the FE of OA at the start of the experiment is nearly 100% in the absence of water. The FE of OA reduced during the course of the experiment to 80% but remained at this value after 5 h. These results clearly show that the catholyte should be water-free to obtain high FEs for OA and limit byproduct formation.

We have also tested the effect of different supporting electrolytes on the electrolysis of CO_2_ to OA. In addition to TEACl, 0.3 M TBAP and 0.5 M TEAA in PC solutions were tested. The results can be found in Figures S7 and S8 of the Supporting Information. Compared to TEACl, the FEs for the systems with TBAP and TEAA are similar, but the CDs and OA concentrations are lower. The lower CDs for the TBAP and TEAA systems are directly related to the lower electrical conductivities of the used mixtures. Note that different electrolyte concentrations were used for TEACl, TEAA, and TBAP due to solubility constraints of the electrolytes in PC. The poor solubility of electrolytes in organic solvents results in a high ohmic resistance in an electrochemical cell. For this reason, a relatively high cell voltage is required to achieve reasonable CDs for CO_2_ electrolysis to OA in nonaqueous media.

**Figure 3:**
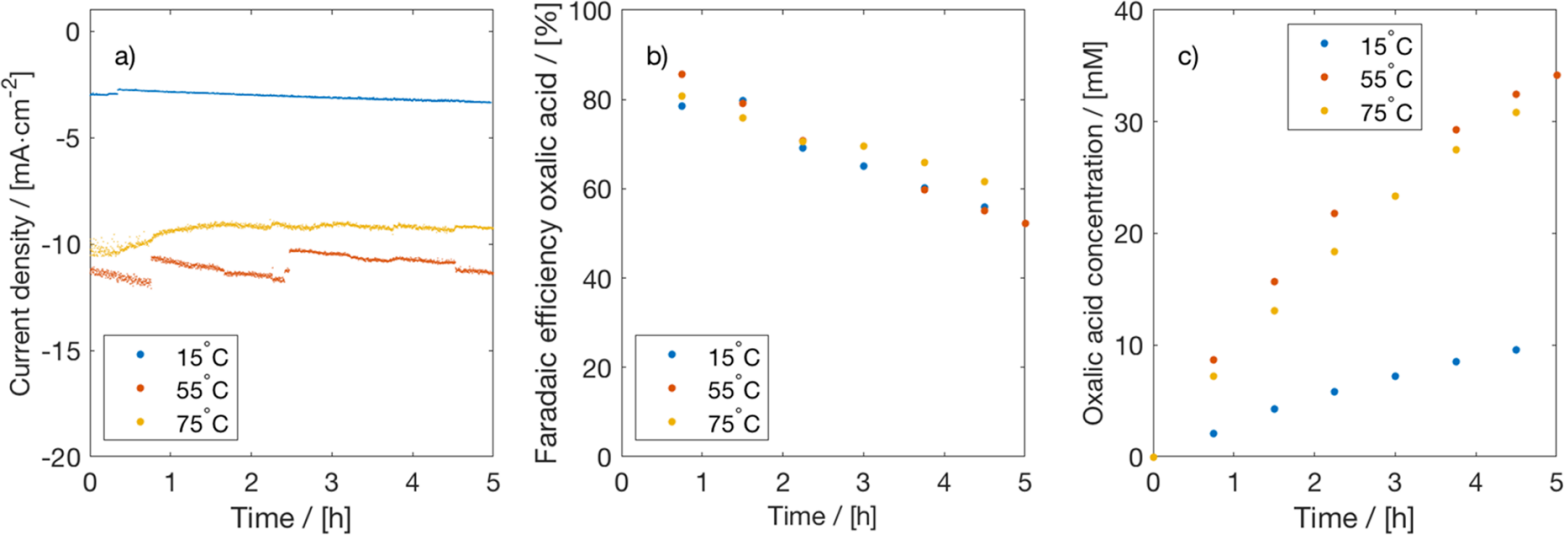
Effect of temperature on CO_2_ electrolysis to oxalate. (a) Current density, (b) Faraday efficiency, and (c) OA concentration for electrochemical reduction of CO_2_ on a Pb cathode in PC with 0.7 M TEACl supporting electrolyte at −2.5 V vs Ag/AgCl in an H-cell at different temperatures. A Pt anode, 0.5 M H_2_SO_4_ as anolyte, and CEM (Nafion 117) were used.

The effect of temperature on CO_2_ conversion to OA in PC was tested in the H-cell. In addition to the experiments at 25 °C reported in Figure 1, CO_2_ electrolysis experiments were performed at 15, 55, and 75 °C in PC with 0.7 M TEACl at −2.5 V vs Ag/AgCl. In Figure 3, a comparison of the results for different temperatures is presented. The FEs of OA are very similar for the three temperatures, but the CDs are higher for higher temperatures. Remarkably, the CD and the OA concentration are the highest for 55 °C. This is due to a competing effect of increased conductivity but decreased CO_2_ solubility at higher temperatures. The low CO_2_ solubility causes mass transfer limitations and results in lower CDs. There are some notable differences in the byproduct distribution as a function of temperature; see Figures S9–S11 of the Supporting Information. At low temperatures, glycolic acid seems to be the major byproduct, while at higher temperatures formic acid is the main byproduct. This can be explained by a higher diffusion rate of water from the anode to the cathode at higher temperatures.

**Figure 4:**
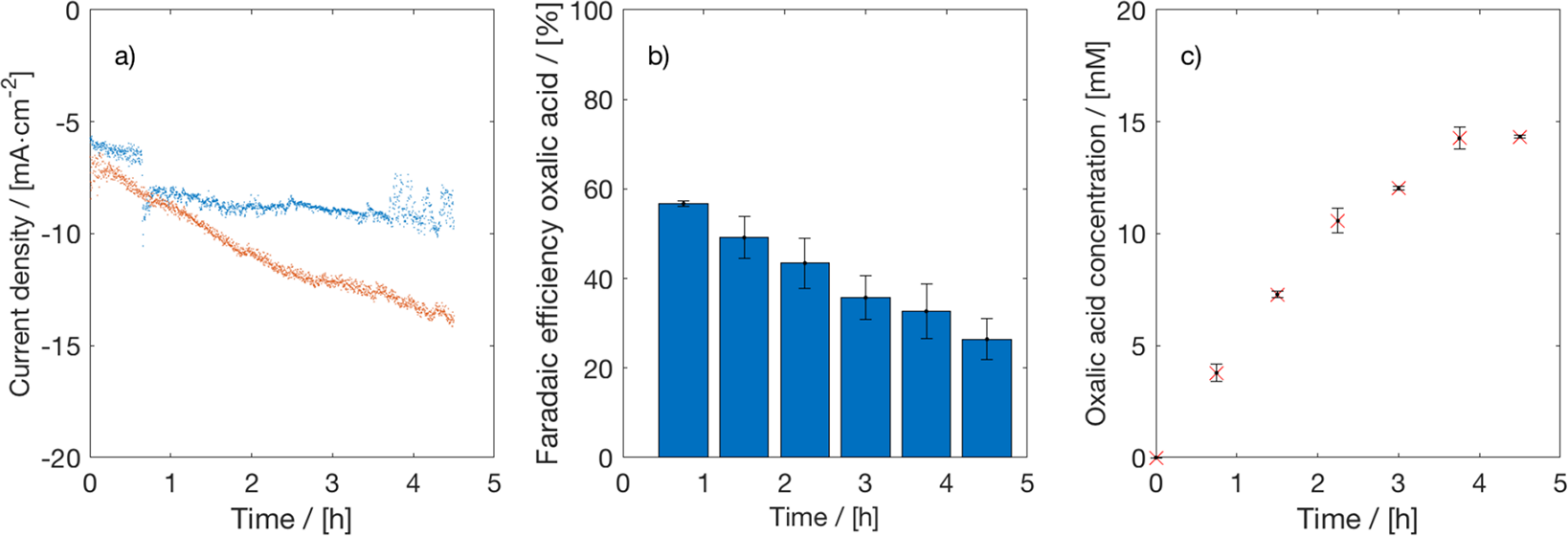
Effect of flow on CO_2_ electrolysis to oxalate. (a) Current density, (b) Faraday efficiency, and (c) OA concentration for electrochemical reduction of CO_2_ on a Pb cathode in PC with 0.7 M TEACl supporting electrolyte at −2.5 V vs Ag/AgCl in a flow cell. A Pt anode, 0.5 M H_2_SO_4_ as anolyte, and CEM (Nafion 117) were used. Duplicate experiments were performed to check reproducibility (blue and orange data).

So far, we have only discussed the results of the H-cell experiments. The best performing conditions of the H-cell experiments were selected for CO_2_ electrolysis to OA in a flow cell. In these experiments, a Pb cathode, Pt anode, 0.5 M H_2_SO_4_ as anolyte, 0.7 M TEACl in PC as the catholyte, and a cation exchange membrane were used. The catholyte and anolyte were both pumped through the cell at a rate of 3.6 L/h/cm^2^. The flow cell experiments were performed at three potentials (−2.3, −2.5, and −2.7 V). In Figure 4, the results of duplicated CO_2_ electrolysis experiments at −2.5 V are presented (see Figures S12 and S13 of the Supporting Information for the results at −2.3 and −2.7 V). In the flow mode, the CDs are slightly higher, but the FEs and the OA concentrations are lower compared to the H-cell experiments. The reduction in the FE can be related to the increased water content of the catholyte as a function of time; see Figure S14 of the Supporting Information. Remarkably, the relative distribution of the byproducts did not change over time; see Figure S15 of the Supporting Information. The FEs of the liquid byproducts are around 10 to 15% throughout the whole experiment with glyoxylic and glycolic acid as the main byproduct. One would expect formic acid as the major byproduct with an increasing water content in the catholyte, but this is apparently not the case here. Clearly, this is different than the H-cell experiments where an increase in the water content of the catholyte resulted in an increased FE of formic acid (see Figure 2).

To conclude, the product distribution in CO_2_ electrolysis to OA strongly depends on the operating conditions such as the CD, potential, water content, CO_2_ concentration, diffusion layer thickness, and type of catholyte and catalyst. Compared to aqueous systems, CO_2_ electrolysis to OA in nonaqueous media presents a range of inherent challenges related to the high overpotentials, water contamination, poor electrolyte solubility, membrane and solvent stability, and catalyst corrosion.

## Process Design and Modeling

**Figure 5:**
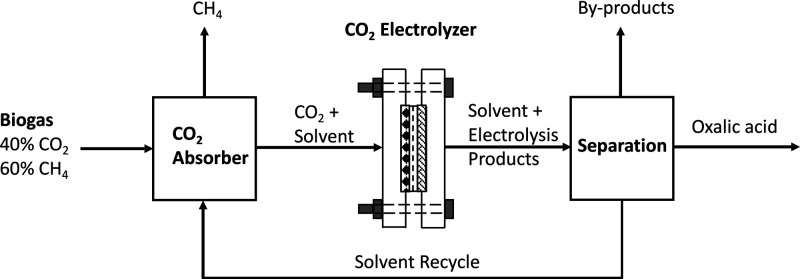
Integrated process for CO_2_ capture, electrochemical conversion, and product separation including solvent recycling.

A schematic of the considered process is shown in Figure 5. The process includes CO_2_ capture, electrochemical CO_2_ conversion, and downstream separation of (by)products, including solvent recycling. CO_2_ is captured from a biogas stream using propylene carbonate, which is a commercial solvent used in the Fluor Solvent Process. In the classical process, the captured CO_2_ would be regenerated from the solvent in a desorber. In our integrated process, the CO_2_ and solvent mixture is sent directly to the CO_2_ electrolyzer (thus eliminating the desorber). In the electrolyzer, CO_2_ is converted to oxalic acid and some byproducts, like glycolic acid and glyoxylic acid, which will be neglected in the base case design. The solvent stream containing the electroreduction products are sent to the separation section where the oxalic acid is recovered. The recovery of oxalic acid/oxalate from nonaqueous solutions is not trivial. The selection of the separation method depends on the pH of the solution, which determines the state of the acid. For the separation, it is important to know whether oxalate or oxalic acid is present in the cathode compartment of the electrolyzer. Note that the state of the product (dissociated or undissociated) depends on the cell configuration. For example, using an undivided cell with a sacrificial zinc anode will produce zinc oxalate as a product. In our experiments, protons from water oxidation in acidic media (i.e., H_2_SO_4_) crossed the CEM and acidified the catholyte (thus producing oxalic acid). To support this hypothesis, we extracted the oxalic acid/oxalate from the organic phase (i.e., PC) into the aqueous phase by simply mixing the catholyte with water and measuring the pH of the aqueous phase. The measured pH was between 1.4 and 1.7, which corresponds well with the expected pH based on the OA concentrations. This confirms that in our experiments mostly OA was produced in the cathode compartment. The protonation of oxalate to OA does not necessarily need to occur on the cathode surface, because this step can equally well happen in the electrolyte.

### CO_2_ Absorption in PC

**Figure 6:**
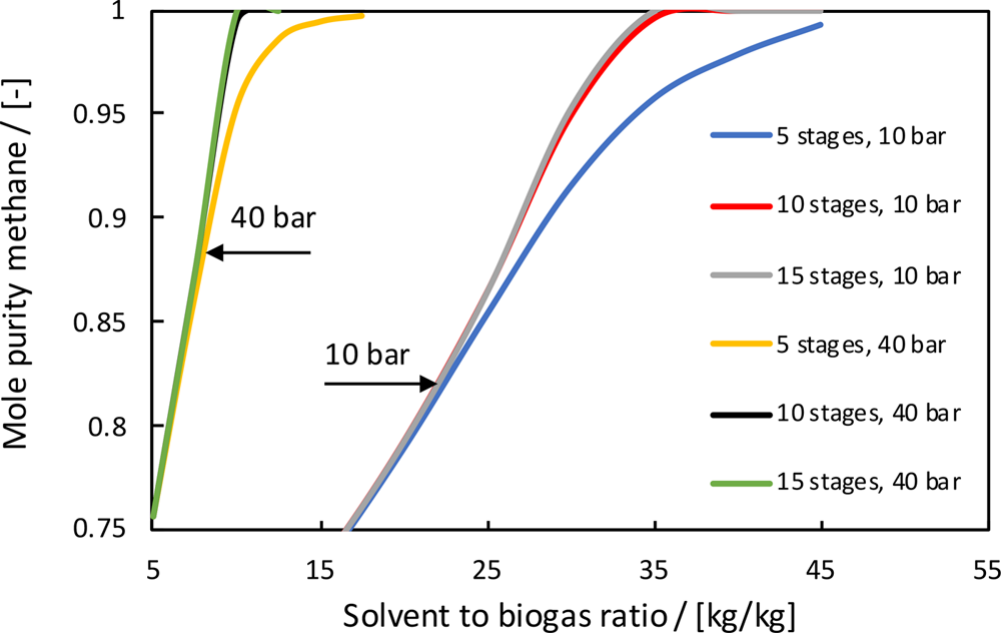
Optimization of the absorption column. The mole purity of methane in the product gas is calculated as a function of solvent flow for different numbers of theoretical stages (5, 10, and 15) and pressures (10 and 40 bar).

The absorption of CO_2_ from biogas with PC as a solvent was modeled in Aspen Plus. We assumed that the feed with a composition of 40 mol % CO_2_ and 60 mol % CH_4_ enters the absorber at 25 °C and 10 bar. The absorber is designed to process 1 ton/h of biogas with a methane purity of at least 94 mol % to comply with the standards for biomethane injection into the natural gas grid of The Netherlands (6 mol % of CO_2_ is allowed).^25^ This means that roughly 90% of the CO_2_ should be removed from the biogas. The solvent flow and the number of stages were varied to meet the design specifications. For the property calculations, the Peng–Robinson (PR) equation of state (EOS) was used. The binary interaction parameters (BIPs) of the PR-EOS were fitted to available experimental solubility data of CO_2_ and CH_4_ in PC; see Table S2 of the Supporting Information. Note that some methane is coabsorbed, which will be carried along with the PC stream to the cathode compartment of the electrolyzer. In Figure 6, the results for the absorber design are shown. The mole purity of methane in the product gas was calculated as a function of the solvent to biogas ratio for different numbers of theoretical stages and two pressures (10 and 40 bar). Operating the column at 40 bar will significantly reduce the solvent flows, but the feed compression costs and the amount of coabsorbed methane will increase. In the process design, we have selected a pressure of 10 bar, 10 stages, and a solvent to biogas ratio of 30 to meet the design specifications.

### Electrochemical Conversion of CO_2_

For the base case, we have assumed that CO_2_ is converted to OA with an FE of 80% at a CD of 100 mA/cm^2^ and cell voltage of 4.0 V. We considered hydrogen as the only byproduct, which is obtained with an FE of 20%. It is assumed that 60% of all dissolved CO_2_ in PC is converted to OA (i.e., conversion of 60% is assumed). The conversion is based on literature data of state-of-the-art CO_2_ electrolyzers.^26^ The electrolyzer is operated at the same pressure as the absorber (10 bar). We assume that the coabsorbed methane is not reduced in the electrolyzer and remains in the liquid phase. The formed H_2_ will mostly escape to the gas phase, since the solubility of H_2_ in PC is very low. The mixture from the electrolyzer is flashed to obtain a gas stream that contains mostly CH_4_ and hydrogen and a liquid stream containing PC, OA, and unconverted CO_2_. The gas stream can be separated into CH_4_ and H_2_ using readily available technologies (e.g., membranes and adsorption), but in our process design we have decided to blend this H_2_/CH_4_ mixture with the methane stream from the absorber and inject it into the natural gas grid. The liquid stream containing PC and OA is subjected to further downstream processing.

### Separation of Oxalic Acid from Nonaqueous Solutions

In principle, several technologies are available for the separation of oxalic acid but mostly from aqueous solutions. We will discuss different separation technologies and select the most promising one for our process based on an elimination procedure. We will see that the state of the acid (dissociated or undissociated) and the requirement of a dry water-free solvent in the electrolyzer have a huge influence on the downstream processing.

#### Liquid–Liquid Extraction

Liquid–liquid extraction (LLE) is a well-established separation technique that is used on an industrial scale, e.g., for formic acid and acetic acid extraction.^27^ In LLE, the solute (OA) is transferred from one liquid phase (feed) to a second liquid phase (extraction solvent), which has a higher affinity for binding the solute. Typically, a water-immiscible solvent is used to extract the solute from an aqueous solution. In our process, the solute (OA) is present in a water-immiscible solvent (PC) and needs to be transferred to another solvent. As briefly explained in the previous section, we have extracted OA from the PC phase using water as the solvent. For the LLE experiments, different amounts of water were added to a PC solution containing 10 mM OA and 0.7 M TEACl and mixed for 48 h. After settling, the concentrations of OA and TEACl in both phases (i.e., the water-rich phase and the PC-rich phase) were measured with HPLC. The distribution coefficient  is defined as
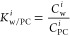
6where  and  are the molar concentrations of component *i* (i.e., OA or TEACl) in the water-rich phase and PC-rich phase. The distribution coefficients of OA and water at 25 °C were 9.6 and 8.6, respectively. In principle, water is a good solvent to extract OA from PC, but a significant amount of TEACl is coextracted as well. The consequence of this is that a second step will be required to separate OA from TEACl, which should be recycled to the electrolyzer. The main problem of the LLE process is that at 25 °C around 7 wt % of water is dissolved in the PC phase, while 17.5 wt % of PC is dissolved in the water phase.^28^ Therefore, the PC phase cannot directly be recycled to the electrolyzer, because the presence of this amount of water would lead to the production of FA and other byproducts (e.g., GCA and GOA). The PC–water mixture cannot simply be distilled due to the presence of a heterogeneous azeotrope. Therefore, a costly dehydration step will be required to dry PC before it can be recycled to the electrolyzer. For this reason, we exclude liquid–liquid extraction with water as a feasible option for OA separation from PC.

#### Electrodialysis

Electrodialysis has been used to purify different types of acids like formic acid, acetic acid, propionic acid, lactic acid, citric acid, and oxalic acid. Wang et al.^29^ used bipolar membrane based electrodialysis (BMED) to convert oxalate from an aqueous solution to oxalic acid. These authors reported an energy consumption of ∼6 kWh/kg for an oxalate concentration of 0.25 mol/L and CD of 30 mA/cm^2^ at 80% current efficiency, but the obtained OA concentration was relatively low. In our process, we cannot use electrodialysis, because the feed contains OA instead of oxalate salt.

#### Crystallization

Crystallization is commonly used in fermentation processes to separate poorly soluble solutes from a solution. In crystallization, the solution is cooled or evaporated beyond the solubility limit of the solute, which then precipitates/crystallizes out. It is clear that solubility data is required to assess the suitability of crystallization for OA crystallization from nonaqueous solvents. In Tables S3 and S4 of the Supporting Information, we provide a compilation of solubility data for OA, GCA, and GOA in water. Unfortunately, solubility data of these acids in nonaqueous solvents is scarce and not available at all for PC. We performed Crystal16 (Technobis) experiments to study the crystallization behavior of OA in PC. In these experiments, the transmission coefficients of 1 M OA samples were measured, while the system was cooled from 60 °C to −10 °C at different cooling rates. The transmission coefficient was close to 100%, which means that no precipitation occurred during the cooling process. For this reason, we exclude cooling crystallization as a potential method for OA separation from PC.

#### Gas Antisolvent Precipitation

Gas antisolvent precipitation (GAP) is a popular method to crystallize pharmaceutical compounds.^30^ In GAP, the solution of an organic solvent containing the product is gradually pressurized with a gas (e.g., supercritical CO_2_), which expands the solution and decreases the solvent power, causing precipitation of the product. The suitability of GAP for OA crystallization depends on the gas–liquid miscibility, the product concentration, and the solubility of OA in PC. We have performed a proof-of-principle experiment to study OA crystallization from PC using GAP with compressed CO_2_ as the antisolvent. Three different solutions of OA in PC (a saturated solution and 0.25 M and 0.5 M solutions) were prepared and loaded into a high pressure sapphire cell; see the Supporting Information for more details of the setup. Next, CO_2_ was gradually added to the cell using a high pressure syringe pump (Teledyne Isco, 260D model). For the saturated solution and 0.5 M solution, precipitation of OA was observed around 30 bar. No precipitation of OA was observed for the 0.25 M OA solution at pressures up to 50 bar. Shishikura et al.^31^ studied OA precipitation from acetone using CO_2_ antisolvent and observed a similar behavior (i.e., OA precipitation occurred only at high concentrations). The GAP process for OA separation from PC seems to work, but only for feeds with sufficiently high OA concentrations. More detailed experiments are required to better understand the precipitation characteristics of OA in nonaqueous solvents. Nevertheless, these preliminary results can be used for conceptual design purposes. We selected the GAP process for the separation of OA from PC.

## Economic Analysis

The profitability of a process can be judged based on different metrics like the payback time (PBT), the return on investment (ROI), or the discounted cash flow or net present value (NPV) approach.^32^ We employed the NPV criteria to evaluate the economic feasibility of the electrochemical reduction of CO_2_ to oxalic acid process. The NPV was calculated by summing the discounted cash flows over the lifetime of the process:
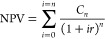
7where *C*_0_ is the initial investment, *C*_*n*_ is the cash flow, *n* is the year, and *ir* is the interest rate. A nominal interest rate of 5% and an income tax rate of 25% was assumed. The straight line depreciation method was applied over a depreciation period of 10 years using a salvage value of 10% of the total capital investment. The working capital was assumed to be 5% of the capital investment, which was recovered at the end of the project. The total CAPEX was obtained as the sum of the capital cost of all process units. The yearly profit was calculated from the revenues generated by selling the products (OA and H_2_) minus the annual OPEX of the process. The value of anodic oxygen and purified methane from the absorber was not considered in the economic analysis. The lifetime of the process was assumed to be 20 years with 8000 h/y of operation.

### Capital Cost Estimation

The capital cost (CAPEX) of the CO_2_ electrolyzer, including the balance of plant (BOP), was taken from our previous work^33^ as $20 000/m^2^. Note that this cost was derived from related electrolysis technologies due to the lack of commercial scale CO_2_ electrolyzers. The required electrolyzer area was calculated from the current density and the set CO_2_ conversion rate. The CAPEX of the CO_2_ absorber was obtained from the Aspen Economic Analyzer after optimizing the number of stages, solvent flow rate, and pressure. The CAPEX of the compressor, which is required to compress the biogas, was obtained from the correlation of Luyben.^34^ The CAPEX of the GAP unit was obtained from a capacity scaling equation:
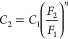
8where *C*_*i*_ is the total battery limit capital cost, *F*_*i*_ is the mass flow of CO_2_ for process *i*, and *n* is the scaling exponent (a value of 0.7 was used here). The reference cost of the GAP unit was taken from Rantakylä^35^ and corrected for inflation using the Chemical Engineering Plant Index (CEPCI) of 2020.^36^ See the Supporting Information for more details of the cost calculations.

### Operating Cost Estimation

The operating cost (OPEX) of the electrolyzer and the compressor was estimated from the power consumption using a base case electricity price of $30/MWh. The power of the electrolyzer is computed from
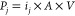
9where *P*_*j*_ is the power required to produce component *j*, *i*_*j*_ is the partial current density for component *i*, *A* is the electrode area, and *V* is the cell voltage. The power of the compressor is obtained from a model for adiabatic compression of an ideal gas; see the Supporting Information. The operating cost of the absorber was directly taken from Aspen Plus using an electricity price of $30/MWh. The power required for pumping the solvent through the reactor is neglected, since this is very small compared to the compression of a gas. Note that the cost of CO_2_ is included in the CAPEX and OPEX of the absorber. The costs of water and recyclable chemicals (e.g., electrolytes and solvents) were neglected in the economic analysis.

### Base Case Assumptions

In Table 1, the data used in the techno-economic analysis for the base case is shown. The parameters of the electrolyzer are based on the latest developments in the field of CO_2_ electrolysis to OA. Thus, the base case data is not necessarily derived from the experiments of this work. A compilation of performance data from recent studies on electrochemical CO_2_ reduction to OA is provided in Table S1 of the Supporting Information. Note that the concentration of OA is limited by the solubility of CO_2_ in PC. At 10 bar and 298.15 K, the solubility is around 0.15 mol CO_2_/mol PC or 1.76 mol CO_2_/L of PC.^37^ This means that, at a CO_2_ conversion of 60%, an OA concentration of only 0.5 M can be obtained in a single pass, since 2 mol of CO_2_ are required per mol of OA. The PC stream with the dissolved OA can be recirculated for higher concentrations, but the concentration cannot be too high to avoid precipitation in the reactor and pipelines. For this reason, we have assumed a concentration of 0.5 M for the base case calculations. The prices of chemicals and electricity are based on the European market. It is important to note that the bulk price of OA in China or India is almost a factor of 2 lower than in Europe. For this reason, the European Union (EU) is imposing an antidumping duty on OA imports from these countries.^38^ The electricity price is based on recent estimates of the U.S. Energy Information Administration for renewable energy from solar and wind.^39^ Most of the base case assumptions are subjected to some uncertainty, which will be taken into account in a sensitivity analysis.

**Table 1:** Base Case Data Used in the Techno-economic Analysis

parameter	value
cell voltage (V)	4
CD (mA/cm^2^)	100
FE (%)	80
CO_2_ conversion (%)	60
concentration OA (M)	0.5
OA price ($/ton)	1000
H_2_ price ($/ton)	1000
electricity price ($/MWh)	30
CAPEX electrolyzer ($/m^2^)	20 000

### Results of the Techno-economic Analysis

**Figure 7:**
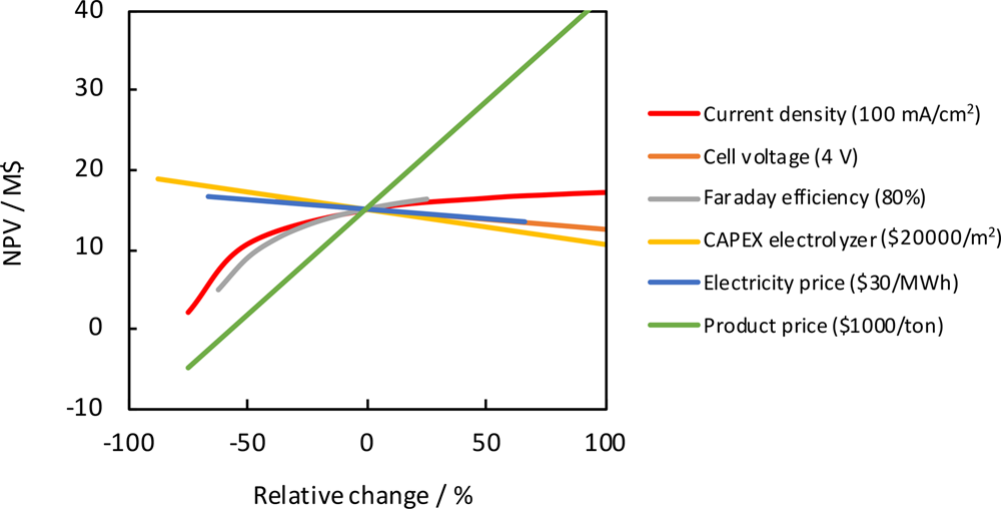
Sensitivity analysis for the economics (NPV) of CO_2_ conversion to OA. Effect of relative changes of the current density, cell voltage, OA Faraday efficiency, electrolyzer CAPEX, electricity price, and product price on the NPV is shown. The base case values are shown in brackets.

In Table 2, the CAPEX and OPEX of the electrochemical CO_2_ conversion process shown in Figure 5 are reported. The total CAPEX and OPEX of the process are roughly $10.7M and $0.3M/y. The CO_2_ electrolyzer accounts for 50% and 75% of the CAPEX and OPEX, respectively. The CAPEX and the OPEX of the downstream separation of OA account for 35% of the total costs. The revenues from selling OA and hydrogen are around $2.9M/y. The sales income of hydrogen is negligibly small compared to OA, since the amount of hydrogen produced is small. The NPV for the base case scenario is positive ($15M), and the PBT is 6 years. These results show that the electrochemical CO_2_ conversion process can be profitable under the base case assumptions. A sensitivity analysis is performed to check the effect of different parameters on the economics of the process. In Figure 7, the results of the sensitivity analysis are shown. Note that only a single input parameter was varied, while keeping other variables constant at the base case values. The relative changes of the input parameters are with respect to the base case values. It is clear that the product price has the largest impact on the NPV. The price of OA should be at least $700/ton to have a positive NPV and a PBT of less than 10 years. As expected, the cell voltage and the electricity price have a similar effect on the economics, since both are related through the power equation. The CD, FE, and electrolyzer CAPEX seems to have a marginal effect on the NPV. It is remarkable that the process has a positive business case for a CD of 50 mA/cm^2^ (NPV of $11M and PBT of 9 years). Electrochemical conversion of CO_2_ to OA seems to have a very favorable economics, which is related to the high market value of OA and the low number of electrons input per mol of product. This can easily be understood by computing the value of 1 mol of supplied electrons:
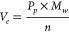
10where *V*_*e*_ is in $/mol electrons, *P*_*p*_ is the market price of the product in $/g, *M*_*w*_ is the molecular weight in (g/mol), and *n* is the moles of electrons required to produce 1 mol of product. The *V*_*e*_ for OA is $0.045/mol of electrons, which is a factor 10 to 15 higher than for ethylene and ethanol.^33^ From a market perspective, OA is the only CO_2_ electroreduction product that can compete with the fossil-based route under the base case scenario.^40,41^ The economics of electrochemical OA production from CO_2_ can be improved even further if higher CDs and FEs and lower cell voltages are achieved and better OA separation methods are developed. Future studies should focus on improving the mass transfer by using gas diffusion electrodes, the elimination of water in the catholyte by, for example, using hydrogen oxidation at the anode, and increasing the electrical conductivity of the solvent/electrolyte mixtures to decrease ohmic losses and the cell voltage.

**Table 2:** Calculated CAPEX and OPEX for CO_2_ Capture, Electrochemical Conversion of CO_2_ to OA, and Downstream Separation

step	CAPEX ($M)	OPEX ($M/y)	CAPEX (%)	OPEX (%)
CO_2_ capture	1.8	0.04	16	12
CO_2_ conversion	5.3	0.26	50	78
OA Separation	3.6	0.03	34	9
total	10.7	0.33	100	100

## Conclusions

We have performed H-cell and flow cell experiments to study the electrochemical reduction of CO_2_ to oxalic acid on a Pb cathode in nonaqueous solvents. The effects of anolyte, catholyte, supporting electrolyte, temperature, catholyte water content, and cathode potential on the FE, CD, and product concentration were investigated. All these parameters influence the performance, but the FE of OA and byproduction formation are mostly affected by the water content of the catholyte. The liquid byproducts glycolic acid, glyoxylic acid, and formic acid are formed in the presence of minor amounts of water. We show that a high FE for OA can be obtained (up to 90%), but the CDs are relatively low (10–20 mA/cm^2^) at a cathode potential of −2.5 V vs Ag/AgCl. A process design and techno-economic evaluation of the value chain for electrochemical conversion of CO_2_ to OA is presented. An integrated process is designed where CO_2_ is captured from biogas (1 ton/h scale) using propylene carbonate, which serves as a nonaqueous solvent in the subsequent step for electrochemical conversion of CO_2_ to OA. It is shown that the requirement of a water-free solvent is significantly complicating the downstream separation of OA from propylene carbonate. We have investigated liquid–liquid extraction, electrodialysis, cooling crystallization, and gas antisolvent precipitation for the downstream separation. The latter process, gas antisolvent precipitation, is the only separation method that seems to work for OA separation from propylene carbonate. An economic analysis of the integrated process, which includes CO_2_ capture, CO_2_ conversion, and downstream separation, is presented. We show that the process has a positive NPV ($15M) and a PBT of 6 years under the base case scenario (CD of 100 mA/cm^2^, OA FE of 80%, cell voltage of 4, electrolyzer CAPEX of $20000/m^2^, electricity price of $30/MWh, and OA price of $1000/ton). A sensitivity analysis shows that the market price of OA has a huge impact on the economics. A market price of at least $700/ton is required to have a positive NPV and a PBT of 10 years. Compared to other CO_2_ electroreduction products, OA has extremely favorable economics due to the relatively high market price and the low number of electrons input per unit of product.

## Supporting Information Available

Data compilation on CO_2_ electrolysis to OA, experimental data on CO_2_ electrolysis at different potentials, temperatures, anolytes, catholytes, and supporting electrolytes, solubility data of OA and GCA in water, details on absorber modeling and GAP experiments, and calculations of capital and operating costs (PDF)
