# Supplementary material for: Electrochemical Reduction of CO2 to Oxalic Acid: Experiments, Process Modeling, and Economics
Source: Ind Eng Chem Res. 2022 Sep 28;61(40):14837–46. doi: 10.1021/acs.iecr.2c02647 (PMC9562277; doi:10.1021/acs.iecr.2c02647)
Supplement: Supplementary file 1 — ie2c02647_si_001.pdf [file ie2c02647_si_001.pdf]

Supporting Information for: Electrochemical Reduction of CO_2 to Oxalic Acid: Experiments, Process Modeling, and Economics

Vera Boor,[†] Jeannine E. B. M. Frijns,[†] Elena Perez-Gallent,[‡] Erwin Giling,[‡] Antero T. Laitinen,[¶] Earl L. V. Goetheer,[‡] Leo J. P. van den Broeke,[†] Ruud Kortlever,[§] Wiebren de Jong,[§] Othonas A. Moultos,[†] Thijs J. H. Vlugt,[†] and Mahinder Ramdin*,[†]

†*Engineering Thermodynamics, Process & Energy Department, Faculty of Mechanical, Maritime and Materials Engineering, Delft University of Technology, Leeghwaterstraat 39, 2628CB Delft, The Netherlands*

‡*Department of Sustainable Process and Energy Systems, TNO, Leeghwaterstraat 44, 2628 CA Delft, The Netherlands*

¶*VTT Technical Research Centre of Finland, Tietotie 4 E, Espoo, Finland*

§*Large-Scale Energy Storage, Process & Energy Department, Faculty of Mechanical, Maritime and Materials Engineering, Delft University of Technology, Leeghwaterstraat 39, 2628CB Delft, The Netherlands*

E-mail: m.ramdin@tudelft.nl

S1 Introduction

This Supporting Information contains the following:

- Table with binary interaction parameters for the Peng-Robinson equation of state
- Tables with solubility data for oxalic acid and glycolic acid in water
- Figures with experimental data on CO_2 electroreduction on Pb cathode in PC with TEACl supporting electrolyte at different potentials in an H-cell.
- Figure with experimental data on CO_2 electroreduction on Pb cathode in acetonitrile with TEACl supporting electrolyte at -2.5 V vs. Ag/AgCl and H_2SO_4 anolyte in an H-cell.
- Figure with experimental data on CO_2 electroreduction on Pb cathode in acetonitrile with TEACl supporting electrolyte at -2.5 V vs. Ag/AgCl and acetonitrile anolyte in an H-cell.
- Figures with experimental data on CO_2 electroreduction on Pb cathode in PC with TBAP and TEAA supporting electrolytes in an H-cell.
- Figures with experimental data on CO_2 electroreduction on Pb cathode in PC with TEACl supporting electrolyte at temperatures in an H-cell.
- Figures with experimental data on CO_2 electroreduction on Pb cathode in PC with TEACl supporting electrolyte at different potentials in a flow cell.
- Figure with water content of the catholyte in the flow cell experiments.
- Figure with experimental data on product distribution for CO_2 electroreduction in the flow cell.
- Description of the high pressure GAP setup

- CAPEX and OPEX calculations for compressors
- CAPEX and OPEX calculations for GAP unit
- CAPEX and OPEX calculations for the absorber from Aspen Plus

Table S1: Compilation of experimental data on CO_2 electroreduction to oxalic acid or oxalate in non-aqueous solvents.

Cell voltage / V	CD / mA/cm^2	FE OA / %	Reference
1.8	10	60	[1]
3	20	80	[1]
5	40	85	[1]
8	60	90	[1]
-	80	90	[1]
-	18	90	[2,3]
-	11	89	[4]
-	20	62	[5]
-	20	45	[6]
3.5	15	77	[7]
-	10	86	[8]
4	80	53	[9]

Table S2: Binary interaction parameters used in the Peng-Robinson EOS modeling. The k_{ij} was fitted to CO_2 and CH_4 solubility data in propylene carbonate.[10,11]

Pair	k_{ij}
CO_2-PC	0.001
CH_4-PC	0.07

Table S3: Solubility (g/100 g) of anhydrous oxalic acid (1) in water (2). Data taken from Riemenschneider et al.[12]

T/ °C	solubility / g/100 g
0	3.5
10	5.5
17.5	8.5
20	9.5
30	14.5
40	22
50	32
60	46
80	85
90	120

Table S4: Solubility (mole fraction) of glycolic acid (1) in water (2). Data taken from Apelblat et al.[13]

T / K	x_1
280.15	0.204
284.05	0.262
288.15	0.316
293.15	0.356
298.15	0.370
303.25	0.388
309.15	0.405
313.15	0.419
321.15	0.438
322.15	0.438
328.15	0.456
332.65	0.466
337.65	0.480
343.15	0.490
348.15	0.501
353.05	0.508
357.65	0.516
361.25	0.521

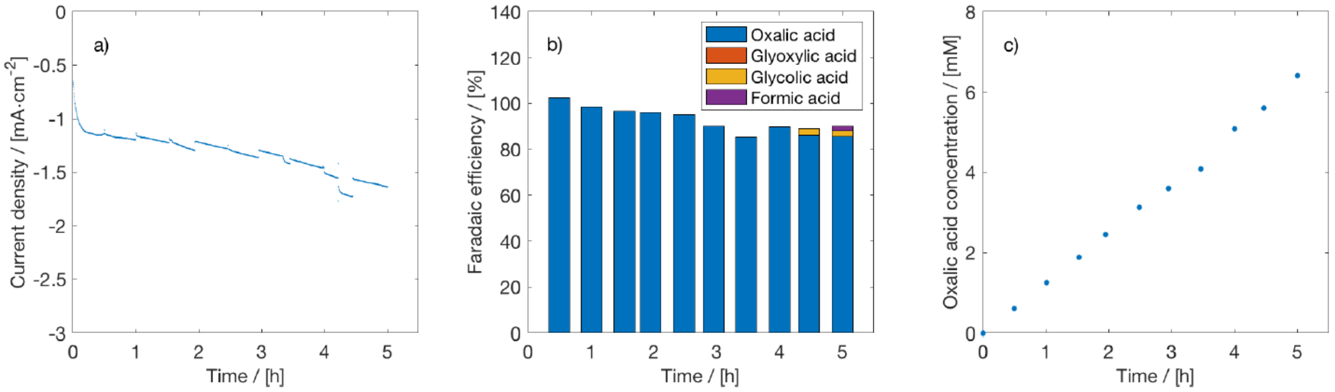

Figure S1: a) Current density, b) Faraday efficiency, and c) OA concentration for electrochemical reduction of CO_2 on a Pb cathode in PC with 0.7M TEACl supporting electrolyte at -2.2 V vs. Ag/AgCl in an H-cell at 298.15 K. A Pt anode, 0.5M H_2SO_4 as anolyte, and CEM (Nafion 117) were used.

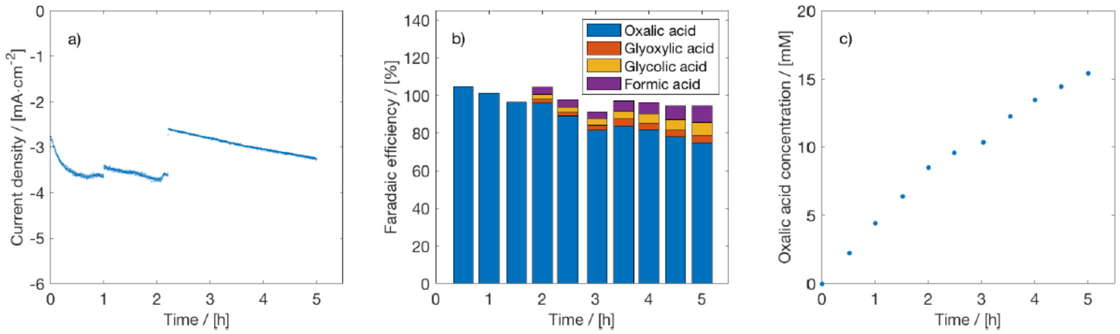

Figure S2: a) Current density, b) Faraday efficiency, and c) OA concentration for electrochemical reduction of CO_2 on a Pb cathode in PC with 0.7M TEACl supporting electrolyte at -2.3 V vs. Ag/AgCl in an H-cell at 298.15 K. A Pt anode, 0.5M H_2SO_4 as anolyte, and CEM (Nafion 117) were used.

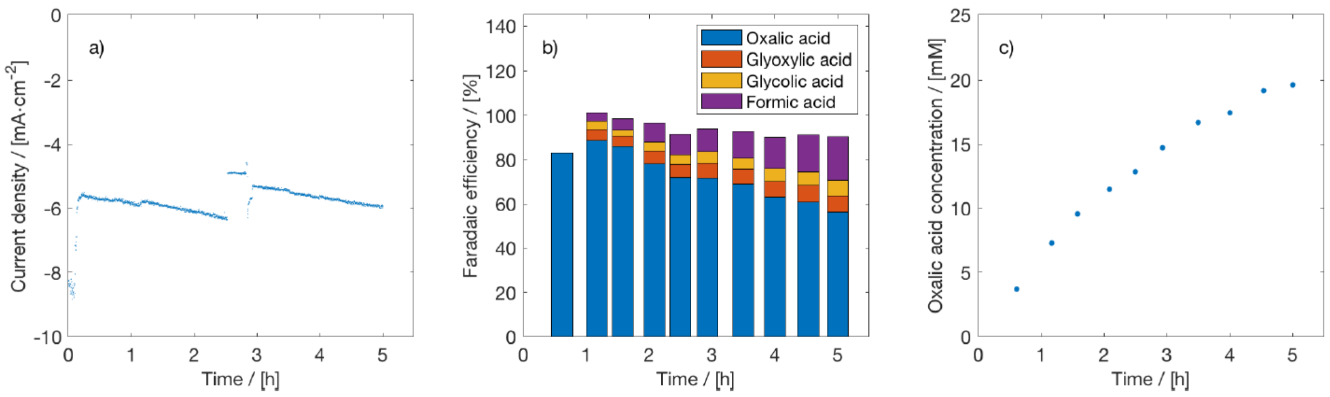

Figure S3: a) Current density, b) Faraday efficiency, and c) OA concentration for electrochemical reduction of CO_2 on a Pb cathode in PC with 0.7M TEACl supporting electrolyte at -2.4 V vs. Ag/AgCl in an H-cell at 298.15 K. A Pt anode, 0.5M H_2SO_4 as analyte, and CEM (Nafion 117) were used.

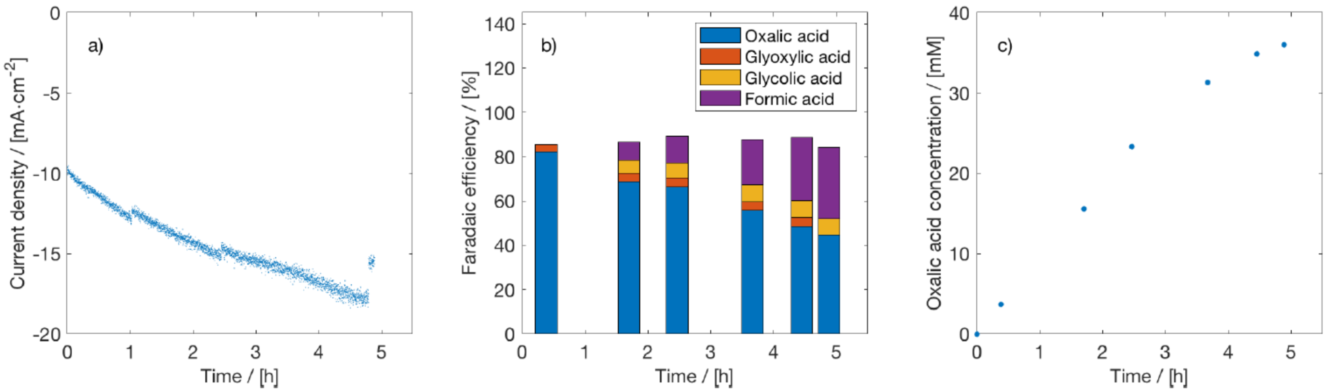

Figure S4: a) Current density, b) Faraday efficiency, and c) OA concentration for electrochemical reduction of CO_2 on a Pb cathode in PC with 0.7M TEACl supporting electrolyte at -2.7 V vs. Ag/AgCl in an H-cell at 298.15 K. A Pt anode, 0.5M H_2SO_4 as analyte, and CEM (Nafion 117) were used.

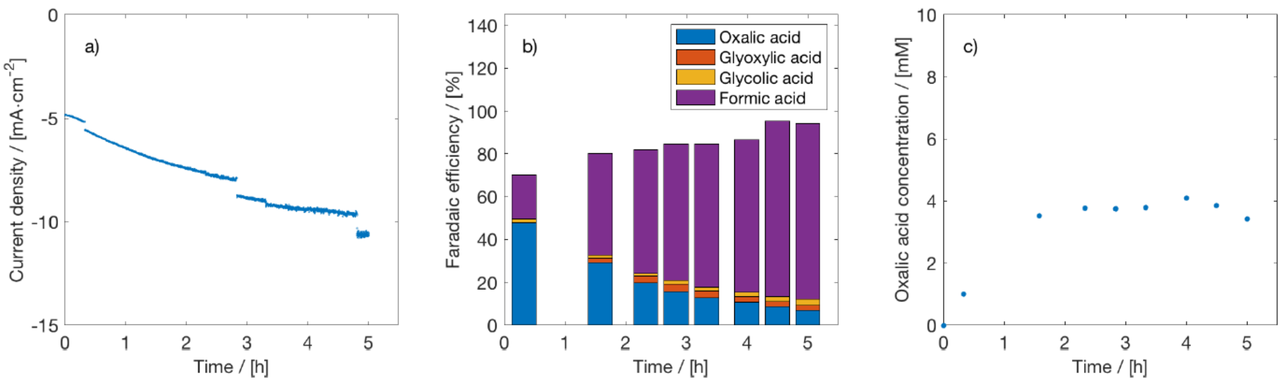

Figure S5: a) Current density, b) Faraday efficiency, and c) OA concentration for electrochemical reduction of CO_2 on a Pb cathode in acetonitrile with 0.1M TEACl supporting electrolyte at -2.5 V vs. Ag/AgCl in an H-cell at 298.15 K. A Pt anode, 0.5M H_2SO_4 as anolyte, and CEM (Nafion 117) were used.

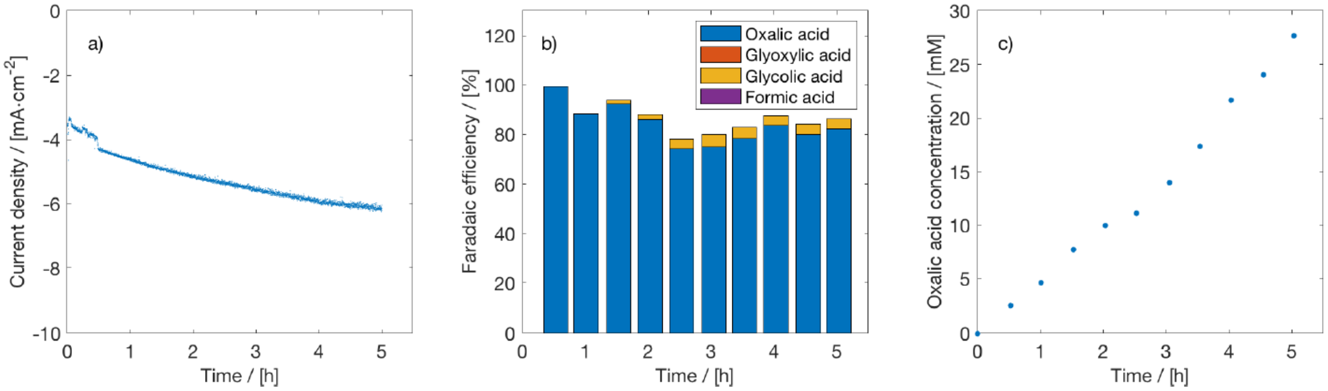

Figure S6: a) Current density, b) Faraday efficiency, and c) OA concentration for electrochemical reduction of CO_2 on a Pb cathode in acetonitrile with 0.1M TEACl supporting electrolyte at -2.5 V vs. Ag/AgCl in an H-cell at 298.15 K. A Pt anode, 0.1M TEACl in acetonitrile as anolyte, and CEM (Nafion 117) were used.

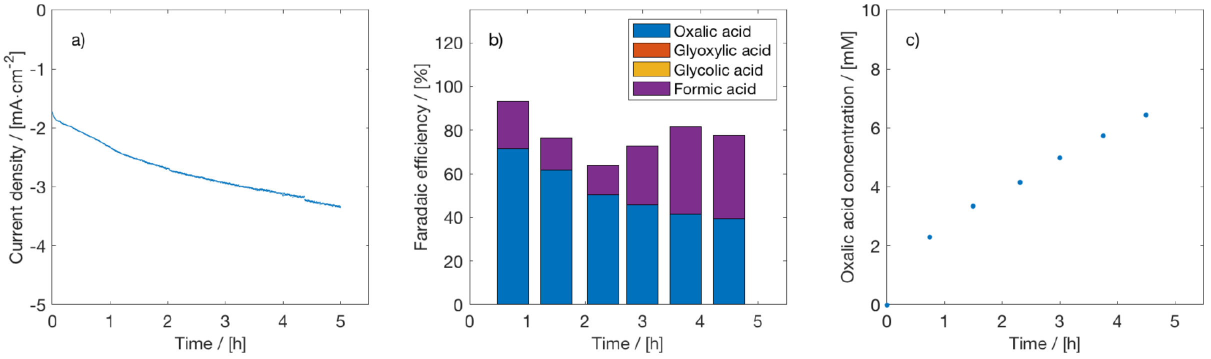

Figure S7: a) Current density, b) Faraday efficiency, and c) OA concentration for electrochemical reduction of CO_2 on a Pb cathode in PC with 0.3M TBAP supporting electrolyte at -2.6 V vs. Ag/AgCl in an H-cell at 298.15 K. A Pt anode, 0.5M H_2SO_4 as analyte, and CEM (Nafion 117) were used.

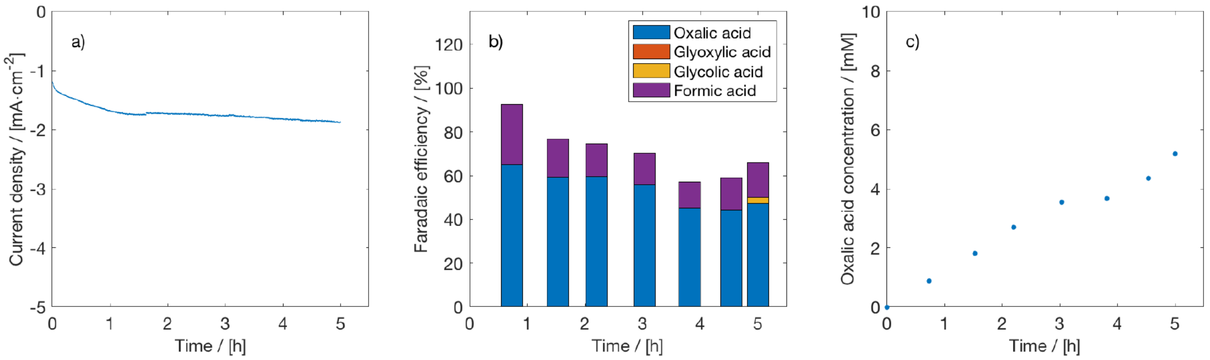

Figure S8: a) Current density, b) Faraday efficiency, and c) OA concentration for electrochemical reduction of CO_2 on a Pb cathode in PC with 0.5M TEAA supporting electrolyte at -2.4 V vs. Ag/AgCl in an H-cell at 298.15 K. A Pt anode, 0.5M H_2SO_4 as analyte, and CEM (Nafion 117) were used.

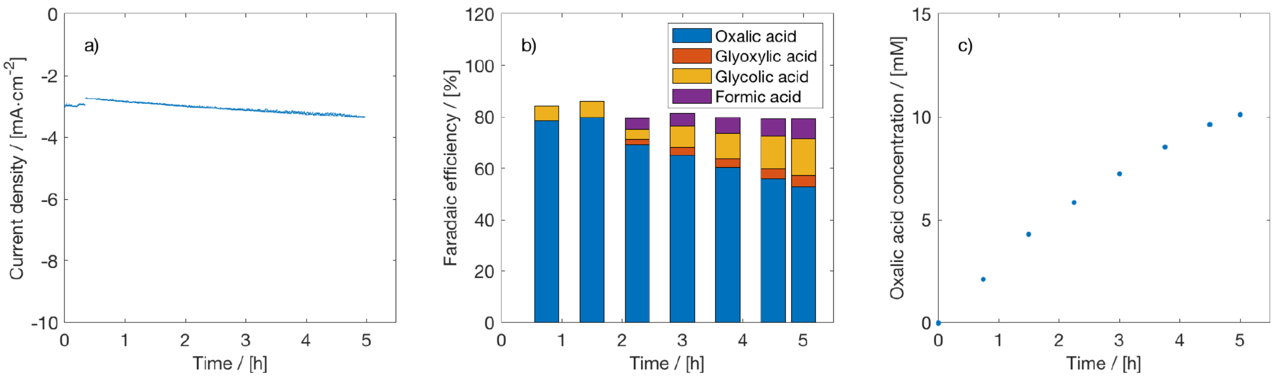

Figure S9: a) Current density, b) Faraday efficiency, and c) OA concentration for electrochemical reduction of CO_2 on a Pb cathode in PC with 0.7M TEACl supporting electrolyte at -2.5 V vs. Ag/AgCl in an H-cell at 15 °C. A Pt anode, 0.5M H_2SO_4 as anolyte, and CEM (Nafion 117) were used.

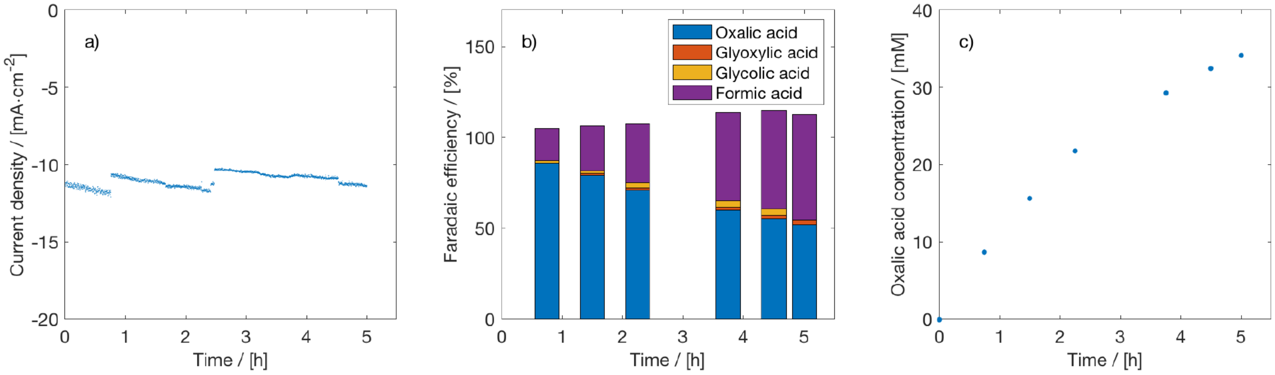

Figure S10: a) Current density, b) Faraday efficiency, and c) OA concentration for electrochemical reduction of CO_2 on a Pb cathode in PC with 0.7M TEACl supporting electrolyte at -2.5 V vs. Ag/AgCl in an H-cell at 55 °C. A Pt anode, 0.5M H_2SO_4 as anolyte, and CEM (Nafion 117) were used.

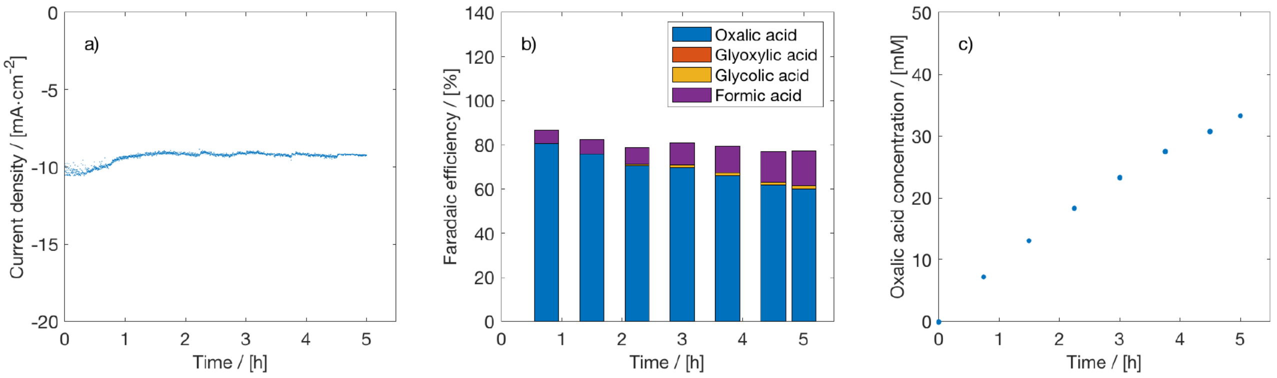

Figure S11: a) Current density, b) Faraday efficiency, and c) OA concentration for electrochemical reduction of CO_2 on a Pb cathode in PC with 0.7M TEACl supporting electrolyte at -2.5 V vs. Ag/AgCl in an H-cell at 75 °C. A Pt anode, 0.5M H_2SO_4 as anolyte, and CEM (Nafion 117) were used.

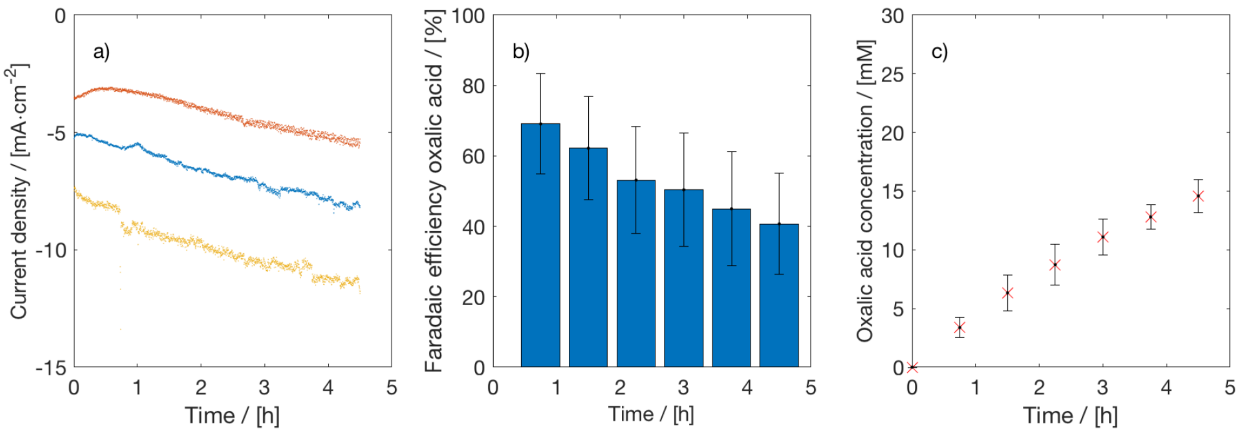

Figure S12: a) Current density, b) Faraday efficiency, and c) OA concentration for electrochemical reduction of CO_2 on a Pb cathode in PC with 0.7M TEACl supporting electrolyte at -2.3 V vs. Ag/AgCl in a flow cell. A Pt anode, 0.5M H_2SO_4 as anolyte, and CEM (Nafion 117) were used. Three experiments were performed to check reproducibility.

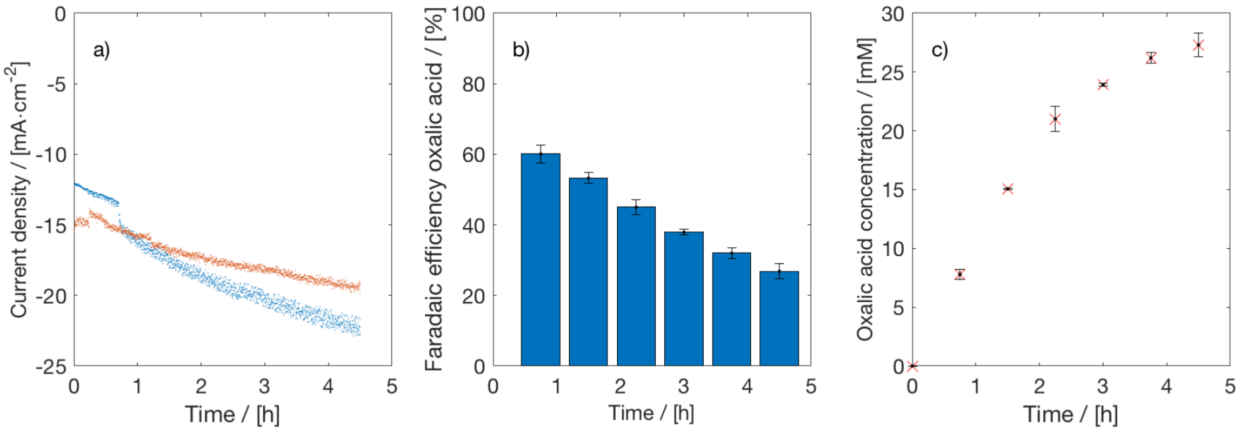

Figure S13: a) Current density, b) Faraday efficiency, and c) OA concentration for electrochemical reduction of CO_2 on a Pb cathode in PC with 0.7M TEACl supporting electrolyte at -2.7 V vs. Ag/AgCl in a flow cell. A Pt anode, 0.5M H_2SO_4 as analyte, and CEM (Nafion 117) were used. Duplicate experiments were performed to check reproducibility.

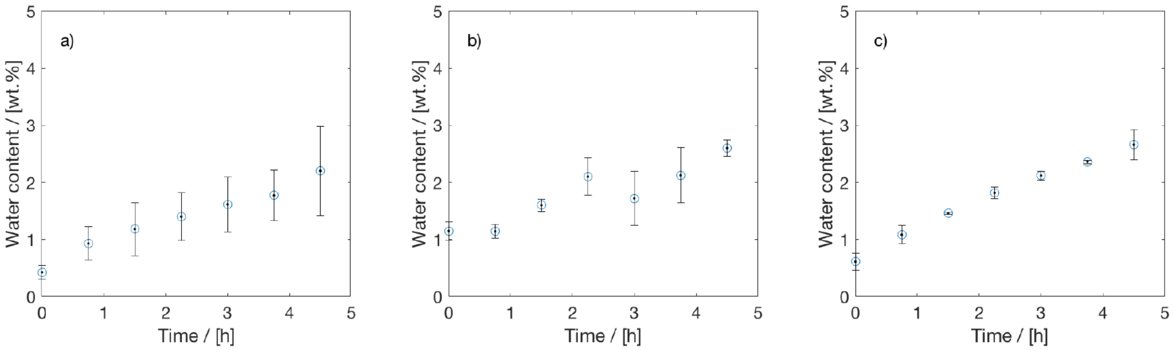

Figure S14: Average water content of the catholyte for the flow-cell experiments performed at a) -2.3 V, b) -2.5 V and c) -2.7 V vs. Ag/AgCl. Experimetns were performed in a flow cell on a Pb cathode in PC with 0.7M TEACl supporting electrolyte. A Pt anode, 0.5M H_2SO_4 as anolyte, and CEM (Nafion 117) were used.

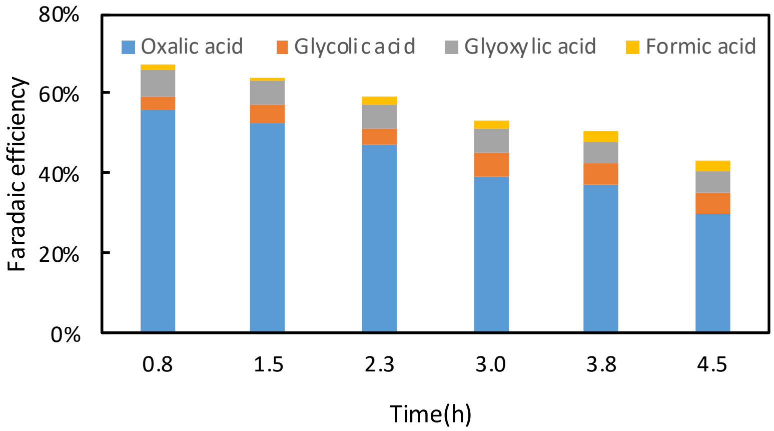

Figure S15: Faraday efficiency for electrochemical reduction of CO_2 on a Pb cathode in PC with 0.7M TEACl supporting electrolyte at -2.5 V vs. Ag/AgCl in a flow cell. A Pt anode, 0.5M H_2SO_4 as anolyte, and CEM (Nafion 117) were used.

S2 High Pressure GAP setup

A simple experimental setup was built to study gas antisolvent precipitation of oxalic acid from propylene carbonate solutions. The setup is shown in Figure S16, which consists of a CO_2 bottle, a high pressure CO_2 pump (Teledyne Isco, 260D model), and a high pressure sapphire cell. The sapphire cell contains a sapphire tube with an outer diameter 0f 4 cm, inner diameter of 1.2 cm, and a length of 8 cm. The tube is enclosed between two flanges, which are held together with four bolts and nuts. The volume of the cell is roughly 10 ml. The sapphire cell can be operated up to a pressure of 200 bar. The cell has no stirrer and temperature control.

The experiments were performed at room temperature by filling the sapphire tube with approximately 3 to 4 ml of oxalic acid solution with a predetermined concentration (saturated, 0.25 M, and 0.5 M in PC). Subsequently, high pressure CO_2 from the bottle via the pump was supplied to the cell. The pressure was controlled by the high pressure pump. After adding CO_2, the cell was given time to reach equilibrium. After, two hours the cell was visually inspected for oxalic acid precipitation.

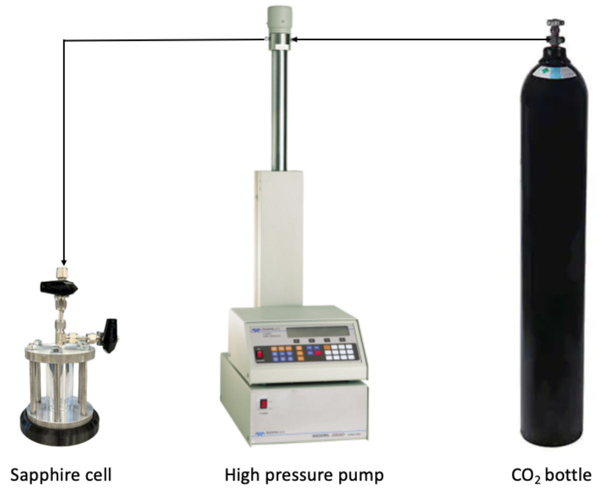

Figure S16: High pressure setup for GAP experiments with CO_2. The setup consists of a CO_2 bottle, a high pressure pump (Teledyne Isco, 260D model), and a high pressure sapphire cell.

S3 CAPEX and OPEX of Compressors

The power input (W) for adiabatic vacuum pumps and compressors for ideal gas can be estimated from:[14]

$$W = (n_f/\eta)\left(\frac{\gamma}{\gamma-1}\right) R * T_1 \left[\left(\frac{P_2}{P_1}\right)^{\frac{\gamma-1}{\gamma}} - 1\right] \tag{S1}$$

where n_f is the mole flow, η is the compressor/pump efficiency assumed to be 0.7, $\gamma = C_P/C_V$ is the adiabatic expansion coefficient, R the ideal gas constant, T_1 the inlet temperature, and (P_2/P_1) is the pressure ratio. We have used $\gamma = 1.4$ for an ideal gas. The inlet temperature and pressure ratio were taken as 298.15 K and 10, respectively.

We process 1 ton/h of biogas, which has a composition of 40 mol% CO_2 and 60 mol% CH_4. This corresponds to 10.2 mol/s of biogas. The calculated power is 117.7 kW. The capital cost of the compressor was estimated from the correlation of Luyben:[15]

$$\text{CAPEX (\$)} = 5840(\text{kW})^{0.82} = 5840(117.7)^{0.82} = \$291334 \tag{S2}$$

The required power (kW) is calculated based on a single stage adiabatic compression assuming an isentropic efficiency of 70%. The operating cost of the compressor is calculated from the electricity price ($0.03/kWh) assuming 8000 h/y of operation.

$$\text{OPEX (\$/y)} = 117.7\text{kW} * 8000\text{h/y} * \$0.03/\text{kWh} = \$28243/\text{y} \tag{S3}$$

S4 CAPEX and OPEX of GAP Unit

The CAPEX of the GAP unit was obtained from a capacity scaling equation:

$$C_2 = C_1 \left(\frac{F_2}{F_1}\right)^n \tag{S4}$$

where C_i is the total battery limit capital cost, F_i the mass flow of CO_2 for process i, and n the scaling exponent (a value of 0.7 was used here). The reference cost of the GAP unit was taken from Rantakyla[16] and corrected for inflation using the Chemical Engineering Plant Index (CEPCI) of 2020. The reference CAPEX was \$2.5M for a CO_2 flow of 1719 kg/h. The minimal flow of CO_2 for our GAP unit is estimated from the solubility of CO_2 in PC at 10 bar, which is roughly 0.15 mol CO_2/mol PC (0.065 g/g). The solvent flow to the absorber is 30 tons/h. The required flow of CO_2 is calculated as: 0.065 (ton/ton) * 30 tons/h = 1.94 tons/h of CO_2. We note that this is a very crude estimation of the CO_2 flow for the GAP unit. Dedicated experiments will be required for more accurate estimation of the CO_2 flow. The capital cost of the GAP unit can now be estimated:

$$\text{CAPEX}_{\text{old}}(\$\text{M}) = 2.5 \left(\frac{1940}{1719}\right)^{0.7} = \$2.7\text{M} \tag{S5}$$

Note that this cost is for the year 2004 (CEPCI = 444.2). We have used the CEPCI of 2020 (596.2) to correct for inflation:

$$\text{CAPEX}_{\text{new}}(\$\text{M}) = \text{CAPEX}_{\text{old}} \left(\frac{596.2}{444.2}\right) = \$3.6\text{M} \tag{S6}$$

The OPEX of the GAP unit is mainly determined by the CO_2 compression cost, which can be calculated as explained in the previous section.

S5 CAPEX and OPEX of Absorber

The absorber was modeled in Aspen Plus using the RADFRAC unit block. The Peng-Robinson EOS was used for the property calculations. The binary interaction parameters were fitted to the experimental solubility data of CO_2 and CH_4 in PC. A comparison of the PR-EOS modeling results and the experimental data is provided in Figure S17. The absorber was designed for 1 ton/h of biogas by calculating the mole purity of methane as a function of the solvent to biogas feed ratio for different number of theoretical stages and pressures. As a design specification, the methane product should have a purity of at least 94%. In the process design, we have selected 10 stages, a solvent to feed ratio of 30, and a pressure of 10 bar. The results of this optimization are provided in the main text. After optimizing the number of stages, pressure, and solvent flow, the CAPEX and OPEX were directly taken from the Aspen Economic Analyzer. The CAPEX was $2.2M, while the OPEX was $15k/y with an electricity price of $30/MWh. We note that the CAPEX from Aspen Plus included a condenser and reboiler, which are absent in our absorber. Therefore, we have a correction factor of 0.8 for the CAPEX.

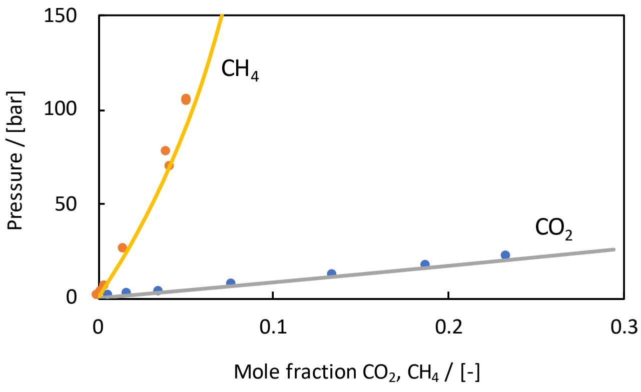

Figure S17: Solubility of CO_2 and CH_2 in propylene carbonate. Points are experimental data[10,11] and lines are PR-EOS modeling results.

References

(1) Fischer, J.; Lehmann, T.; Heitz, E. The production of oxalic acid from CO_2 and H_2O. *J. Appl. Electrochem.* **1981**, *11*, 743–750.

(2) Tyssee, D.; Wagenknecht, J.; Baizer, M.; Chruma, J. Some cathodic organic syntheses involving carbon dioxide. *Tetrahedron Lett.* **1972**, *13*, 4809–4812.

(3) Kaiser, U.; Heitz, E. Zum Mechanismus der elektrochemischen Dimerisierung von CO_2 zu Oxalsäure. *Berichte der Bunsengesellschaft für Phys. Chemie* **1973**, *77*, 818–823.

(4) Lv, W. X.; Zhang, R.; Gao, P. R.; Gong, C. X.; Lei, L. X. Electrochemical Reduction of Carbon Dioxide on Stainless Steel Electrode in Acetonitrile. *Adv. Mater. Res.* **2013**, *807-809*, 1322–1325.

(5) Lv, W.; Zhang, R.; Gao, P.; Gong, C.; Lei, L. Electrochemical reduction of carbon dioxide with lead cathode and zinc anode in dry acetonitrile solution. *J. Solid State Electrochem.* **2013**, *17*, 2789–2794.

(6) Oh, Y.; Vrubel, H.; Guidoux, S.; Hu, X. Electrochemical reduction of CO_2 in organic solvents catalyzed by MoO_2. *Chem. Commun.* **2014**, *50*, 3878.

(7) Subramanian, S.; Athira, K.; Anbu Kulandainathan, M.; Senthil Kumar, S.; Barik, R. New insights into the electrochemical conversion of CO2 to oxalate at stainless steel 304L cathode. *J. CO2 Util.* **2020**, *36*, 105–115.

(8) Yang, Y.; Gao, H.; Feng, J.; Zeng, S.; Liu, L.; Liu, L.; Ren, B.; Li, T.; Zhang, S.; Zhang, X. Aromatic Ester-Functionalized Ionic Liquid for Highly Efficient CO_2 Electrochemical Reduction to Oxalic Acid. *ChemSusChem* **2020**, *13*, 4900–4905.

(9) König, M.; Lin, S.-H.; Vaes, J.; Pant, D.; Klemm, E. Integration of aprotic CO_2 reduction to oxalate at a Pb catalyst into a GDE flow cell configuration. *Faraday Discuss.* **2021**, *230*, 360–374.

(10) Murrieta-Guevara, F.; Romero-Martinez, A.; Trejo, A. Solubilities of carbon dioxide and hydrogen sulfide in propylene carbonate, N-methylpyrrolidone and sulfolane. *Fluid Phase Equilib.* **1988**, *44*, 105–115.

(11) Jou, F.-Y.; Mather, A. E.; Schmidt, K. A. G. Solubility of Methane in Propylene Carbonate. *J. Chem. Eng. Data* **2015**, *60*, 1010–1013.

(12) Riemenschneider, W.; Tanifuji, M. *Ullmann's Encycl. Ind. Chem.*; Wiley-VCH Verlag GmbH & Co. KGaA: Weinheim, Germany, 2011; pp 6–9.

(13) Apelblat, A.; Manzurola, E. Solubility of suberic, azelaic, levulinic, glycolic, and diglycolic acids in water from 278.25 K to 361.35 K. *J. Chem. Thermodyn.* **1990**, *22*, 289–292.

(14) Barecka, M. H.; Ager, J. W.; Lapkin, A. A. Economically viable CO_2 electroreduction embedded within ethylene oxide manufacturing. *Energy Environ. Sci.* **2021**, *14*, 1530–1543.

(15) Luyben, W. L. Capital cost of compressors for conceptual design. *Chem. Eng. and Process.: Process Intensif.* **2018**, *126*, 206–209.

(16) Rantakylä, M. *Particle Production By Supercritical Antisolvent Processing Techniques*; 2004; p 127.
